# Cataract progression after primary pars plana vitrectomy for uncomplicated rhegmatogenous retinal detachments in young adults

**DOI:** 10.1186/s40942-024-00538-4

**Published:** 2024-02-21

**Authors:** Venkatkrish M. Kasetty, Pedro F. Monsalve, Dhruv Sethi, Candice Yousif, Thomas Hessburg, Nitin Kumar, Abdualrahman E. Hamad, Uday R. Desai

**Affiliations:** 1 Department of Ophthalmology, Henry Ford Health System, Detroit, MI USA; 2 Department of Ophthalmology, University of Minnesota, Minneapolis, MN USA

**Keywords:** Rhegmatogenous retinal detachment, Vitrectomy, Cataract

## Abstract

**Background:**

Scleral buckling is typically implemented to repair rhegmatogenous retinal detachments (RRD) in young patients. Therefore, there is limited data on post-pars plana vitrectomy (PPV) cataract formation in this cohort. We report the rates and risk factors of cataract progression after PPV for RRD repair in young eyes.

**Methods:**

Retrospective single-center cohort study. Medical records of patients between the ages of 15 to 45 undergoing PPV for uncomplicated RRD between 2014 and 2020 were reviewed.

**Results:**

Twenty-eight eyes from 26 patients met inclusion criteria. Cataracts developed in 20/28 (71%) eyes after PPV. After PPV, nuclear sclerotic cataract (NSC) rates were higher in patients above 35 (65%) compared to below 35 years (18%) (*p* = 0.024). Cataracts developed more frequently after macula-off RRDs (88%) compared to macula-on RRDs (50%) (*p* = 0.044) with NSC more common in macula-off detachments (*p* = 0.020). At postoperative month 2, all eyes with C_3_F_8_ gas developed cataracts compared to 59% of eyes with no gas (*p* = 0.040).

**Conclusions:**

Cataract formation was common and frequent after PPV. After PPV, young eyes and macula-on detachments developed cataracts less frequently than older eyes and macula-off detachments. If appropriate, a shorter acting gas tamponade should be considered in young eyes to minimize cataract formation.

**Supplementary Information:**

The online version contains supplementary material available at 10.1186/s40942-024-00538-4.

## Background

Rhegmatogenous retinal detachments (RRD) occur in a bimodal distribution affecting the elderly as well as younger, typically myopic patients [1]. Various repair modalities exist including pars plana vitrectomy (PPV), scleral buckling (SB), and pneumatic retinopexy. PPV has been shown to increase the rate of nuclear sclerotic cataracts (NSC) and posterior subcapsular cataracts (PSC) formation with minimal lens changes seen after SB. Increased oxygen tension after vitreous removal, prolonged exposure to saline solution during PPV, use of higher purity and longer longevity gases, direct trauma to the lens, and larger vitrectomy gauges have been proposed as causes of increased cataract formation after PPV; however, the mechanism is still unclear [2, 3]. Analysis on post-PPV cataract formation is limited in young eyes as SB is usually preferred in this group [4–6]. At our institution, some vitreoretinal surgeons prefer PPV over SB in this age group. While prior studies have evaluated the rates and risk factors associated with cataract progression after PPV in all ages, we focus our analysis on young patients to determine the rates of and risk factors for cataract progression.

## Methods

This is a retrospective cohort study of patients ages 15 to 45 years old with no prior retinal surgery undergoing PPV for RRD repair between 2014 and 2020 at Henry Ford Health System in Michigan, USA. Primary PPV was chosen for these patients if they had a pre-operative PVD or if the vitreoretinal surgeon felt that they could induce a PVD intraoperatively. Ethical approval for this study was obtained from the Henry Ford Health System Institutional Review Board. This study adhered to the tenets of the Declaration of Helsinki. Due to the retrospective nature of the study, written informed consent was not required. Exclusion criteria included pseudophakic or aphakic eyes, eyes with less than 3 months of follow-up, eyes requiring lensectomy at the time of initial RRD repair, and combined detachments (diabetic, inflammatory, and infectious etiologies). Only eyes undergoing one surgery for RRD repair were primarily analyzed to avoid the effects of reoperations on cataract formation. Eyes with mild preoperative cataracts were included in the analysis.

Primary outcomes of this study include cataract formation rates after RRD repair and time to cataract extraction from initial PPV. Cataract formation was recorded based on type: NSC, PSC, and cortical. Cataract severity was recorded pre-operatively, at post-operative month 1 (POM1), post-operative month 3 (POM3), and post-operative year 1 (POY1). Cataract grading was based on the Lens Opacities Classification System, version II (LOCS II) scale of 1–4 (with 4 being the worst) [7]. Secondary outcomes include changes in pre- and post-cataract surgery visual acuity (VA) and correlations between age, preoperative refraction, posterior vitreous detachment (PVD) presence, macula status, surgical time and cataract development or progression. As most retinal detachments have stable VA 3–6 months after repair, postoperative month 3 was selected to minimize effects of intraocular gas and postoperative cataracts on VA in our analysis [8]. 

Myopia is defined as a refraction between − 6.0 and 0 diopters (D). High myopia is defined as a refraction greater than − 6.0 D. Surgical time was used was a surrogate for saline infusion time during PPV.

Fisher’s exact test was performed for categorical covariates and Mann-Whitney U tests were performed for numeric covariates. All analyses were performed using RStudio statistical software (RStudio, Boston, Massachusetts, USA).

## Results

Twenty-eight eyes (26 patients) undergoing PPV were included in this study. Initial PPV was performed by one of three vitreoretinal surgeons. The average age at PPV was 35.39 ± 8.26 years with 20/28 (71%) female. Refraction was available for 23/28 (82%) eyes, of which 8/23 (35%) and 11/23 (48%) were myopic and highly myopic, respectively. One patient (1 eye) had a diagnosis of Wagner-Stickler Syndrome and 2 patients (3 eyes) had type 2 diabetes mellitus without retinopathy. A posterior vitreous detachment (PVD) was present in 18/28 (64%) eyes with 16/28 (57%) eyes presenting with a macula-off detachment. The crystalline lens was clear in all but one eye, which had a mild cataract. Standard 23-gauge vitrectomy was performed in all vitrectomized eyes. Perfluoropropane (C_3_F_8_) gas was used in all but one vitrectomized eye where sulfur hexafluoride (SF_6_) gas was used instead. There were no documented lens touch with instrumentation during PPV. The average surgical time was 69 ± 27 min. Average follow-up length was 859 ± 800 days with all retinas attached at last follow-up. One eye had pre-operative proliferative vitreoretinopathy (PVR) and no eyes developed PVR after PPV. No eyes were enucleated.

Cataracts developed in 20/28 (71%) eyes after PPV with 17/28 (61%) developing within 1 year. NSC, PSC, and cortical cataracts developed in 13/28 (46%), 12/28 (43%), and 2/28 (7%) eyes, respectively, after PPV. Average cataract grading at each post-operative visit is presented in Table 1. The average NSC and PSC grading increased in PPV eyes through 1 year. NSC grading at POM1 (*p* = 0.021), POM3 (*p* = 0.011), and POY1 (*p* = 0.017) were significantly greater compared to pre-operative NSC grading. However, PSC grading at POM1 (*p* = 0.021), POM3 (*p* = 0.005), but not POY1 (*p* = 0.079) were significantly greater compared to pre-operative PSC grading. On average, cataracts developed 180 ± 308 days after PPV. Eleven (39%) eyes required cataract extraction, which occurred 375 ± 212 days after PPV. The average age at PPV of eyes requiring cataract extraction was 38.27 ± 8.56 years compared to 33.52 ± 7.74 years of eyes not requiring cataract extraction after PPV (*p* = 0.085).

**Table 1:** Average cataract grading after pars plana vitrectomy

		Pre-Operative	POM1	POM3	POY1
**NSC**	**Grade**	0.04	0.30	0.51	0.46
	***p-value***	-	0.021*	0.011*	0.017*
**PSC**	**Grade**	0.00	0.44	0.45	0.25
	***p-value***	-	0.021*	0.005*	0.079

Key: NSC, nuclear sclerotic cataract; PSC, posterior subcapsular cataract; POM1, post-operative month 1; POM3, post-operative month 3; POY1, post-operative year one. * indicates statistically significant *p*-value when compared to pre-operative cataract grading

**Fig. 1:**
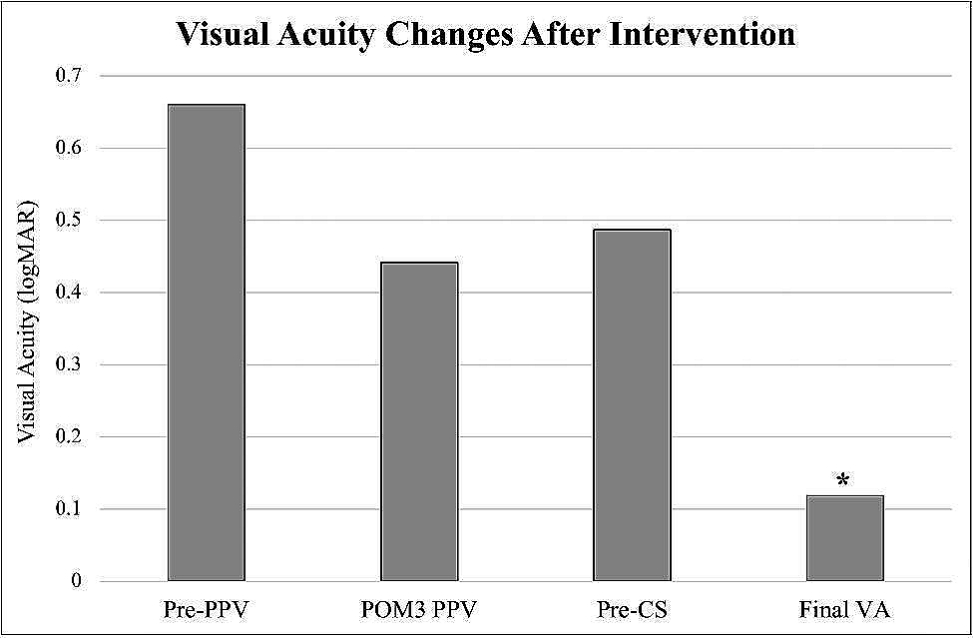
Visual Acuity (VA) Changes after Intervention. After retinal reattachment, there was no significant improvement in VA compared to before PPV (*p* = 0.513). There was a slight decrease in VA between POM3 and pre-CS (*p* = 0.856). VA significantly improved after cataract surgery compared to before cataract surgery (*p* = 0.006). ^*^Denotes statistical significance between last recorded VA and pre-cataract surgery VA in eyes undergoing PPV. Abbreviations: CS, cataract surgery; POM3, postoperative month 3; PPV, pars plana vitrectomy

VA changes over time are presented in Fig. 1. After PPV, average VA improved to 20/50 from 20/100 (*p* = 0.513). There was a slight decrease in average VA from 20/50 to 20/60 when comparing three months after PPV to before cataract extraction (*p* = 0.856). The average time to cataract extraction was 375 days (range: 109–772) after PPV with 6/11 (55%) extractions occurring within 1 year of PPV. Significant VA gains were seen after cataract surgery with an improvement from 20/60 to 20/25 at the last recorded visit (*p* = 0.006).

Cataracts developed in 7/11 (64%) eyes in patients below 35 years compared to 13/17 (76%) of eyes in patients above 35 years (*p* = 0.672) (Table 2). Patients above 35 years developed NSC (11/17, 65%) more frequently than patients below 35 years (2/11, 18%) (*p* = 0.024). However, patients below 35 years developed PSC at similar rates (6/11, 54%) compared to patients above 35 years (7/17, 41%) (*p* = 0.700). Of eyes developing cataracts, 3/7 (43%) eyes below 35 years and 8/13 (62%) above 35 years required cataract extraction (*p* = 0.624). Cataracts also developed sooner in patients over 35 years (110 ± 147 days) compared to under 35 years (300 ± 467 days) but was not statistically significant (*p* = 0.140).

**Table 2:** Risk factors for cataract progression after PPV

		Cataract Rate	NSC Rate	PSC Rate	Time to Cataract Development (Days)
**Age**	< 35 years old	7/11 (64%)	2/11 (18%)	6/11 (54%)	300 ± 467
	≥ 35 years old	13/17 (76%)	11/17 (65%)	7/17 (41%)	110 ± 147
	*p*-value	0.672	0.024*	0.700	0.140
**Refraction**	Myopia	6/8 (75%)	5/8 (63%)	4/8 (50%)	139 ± 99
	High myopia	8/11 (73%)	5/11 (45%)	4/11 (36%)	244 ± 470
	*p*-value	1.00	0.645	0.658	0.181
**PVD**	Present	14/18 (78%)	9/18 (50%)	10/18 (56%)	230 ± 380
	Absent	6/10 (60%)	4/10 (40%)	4/10 (40%)	94 ± 71
	*p*-value	0.4	0.695	0.706	0.902
**Macula Status**	On	6/12 (50%)	3/12 (25%)	3/12 (25%)	324 ± 507
	Off	14/16 (88%)	12/16 (75%)	9/16 (56%)	114 ± 140
	*p*-value	0.044*	0.020*	0.136	0.412
**Surgical Time**	< 60 min	9/13 (69%)	5/13 (38%)	5/13 (38%)	288 ± 456
	≥ 60 min	11/15 (73%)	8/15 (53%)	7/15 (43%)	102 ± 94
	*p*-value	1.00	0.476	0.712	0.840

Key: NSC, nuclear sclerotic cataract; PSC, posterior subcapsular cataract; PPV, pars plana vitrectomy; PVD, posterior vitreous detachment. * denotes a statistically significant *p*-value

Cataracts developed at higher rates after macula-off RRDs (14/16, 88%) compared to macula-on RRDs (6/12, 50%) (*p* = 0.044) (Table 2). Specifically, NSC developed more frequently in macula-off detachments (12/16, 75%) compared to macula-on detachments (3/12, 25%) (*p* = 0.020). There was no significant difference in PSC rates between macula-on and macula-off RRDs (*p* = 0.136). Cataracts also developed sooner after macula-off RRD (114 ± 140 days) compared to macula-on RRD (324 ± 507 days) but was not statistically significant (*p* = 0.412).

Eyes developing cataracts had an increased postoperative gas fill at all time points compared to eyes without cataracts; however, this difference was not statistically significant at any time point (Table 3). Cataracts developed in 8/8 (100%) eyes with residual C_3_F_8_ at postoperative month 2 compared to only 11/18 (61%) eyes without residual C_3_F_8_ at postoperative month 2 (*p* = 0.047). The eye with an SF_6_ gas tamponade did not develop a cataract.

**Table 3:** Post-PPV C_3_F_8_ Fill

	POD1 C_3_F_8_ (%)	POW1 C_3_F_8_ (%)	POM1 C_3_F_8_ (%)	POM2 C_3_F_8_ (%)
**Cataract**	88.3	71.6	34.5	5.8
**No Cataract**	80.7	65.9	22.6	0

Key: POD1, postoperative day 1; POM1, postoperative month 1; POM2, postoperative month 2; POW1, postoperative week 1; PPV, pars plana vitrectomy

Other risk factors for cataract development, such as myopic status, PVD presence, and surgical time was not associated with increased cataract formation rates or increased time to cataract formation (Table 2). Myopic eyes developed NSC and PSC at similar rates of 5/8 (63%) and 4/8 (50%) compared to highly myopic eyes with rates of 5/11 (45%) and 4/11 (36%), respectively (*p* = 0.645 and 0.658). Of eyes with a preoperative PVD, 14/18 (78%) developed cataracts compared to 6/10 (60%) eyes without preoperative PVD (*p* = 0.4). PSC and NSC occurred at similar rates in eyes with a PVD (56% and 50%, respectively) compared to eyes without a PVD (40% each) (*p* = 0.706 and 0.695). Surgical time was not associated with increased cataract formation (*p* = 1.000). While NSC and PSC occurred more frequently in PPV taking over 60 min (53% and 43%) compared to below 60 min (38% and 38%), there was no significant difference between the groups (*p* = 0.476 and 0.712). While there were no documented cases of instrument-lens touch, in eyes that may have required crossing the anatomic midline with instrumentation to treat the breaks, 11/16 (69%) developed cataracts. In eyes that did not require crossing the anatomic midline with instrumentation to treat the breaks, 9/12 (75%) developed cataracts (*p* = 1.00). Additionally, intraoperative use of intravitreal triamcinolone acetonide (IVT) to stain the posterior hyaloid did not result in increased cataract formation. When IVT was used, 6/10 (60%) of eyes developed cataracts compared to 14/18 (78%) when IVT was not used (*p* = 0.400). PSC occurred in 4/10 (40%) and 8/18 (44%) eyes with and without IVT use, respectively (*p* = 1.00). NSC occurred less frequently when IVT was used 2/10 (20%) compared to when IVT was used 11/18 (61%), but was not statistically significant (*p* = 0.055).

**Fig. 2:**
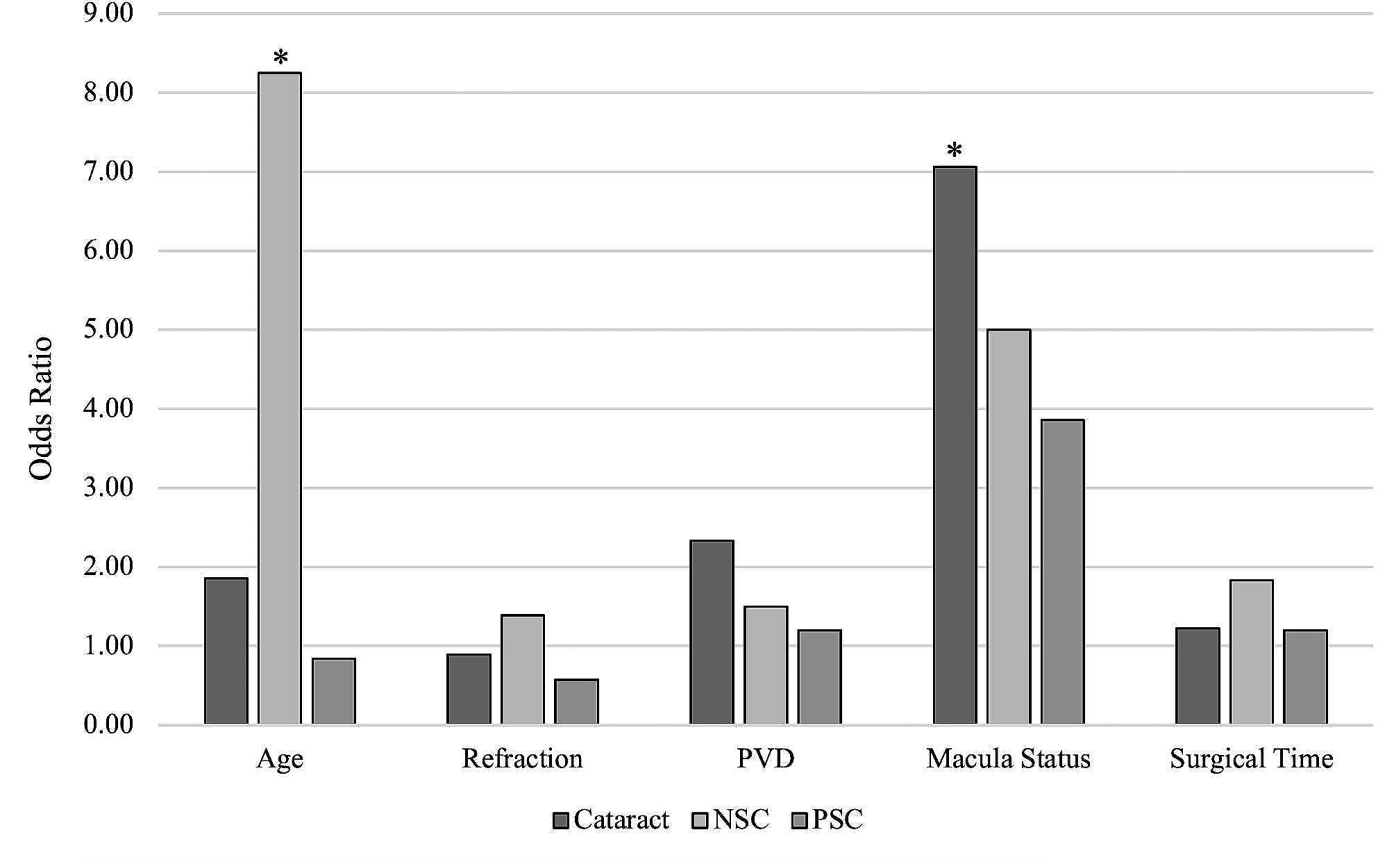
Logistic Regression Analysis of Risk Factors Associated with Cataract Formation after PPV. ^*^Denotes statistically significant odds ratio. Abbreviations: PVD, posterior vitreous detachment; NSC, nuclear sclerosis cataract; PSC; posterior subcapsular cataract

Univariate logistic regression analysis is presented in Table 4; Fig. 2. Similar to the non-parametric analysis above, regression analysis revealed a statistically significant correlation between macula status and overall cataract formation as well as age and NSC formation. Regression analysis did not reveal a significant association between other risk factors and cataract formation.

**Table 4:** Odds Ratios of Risk Factors Associated with Cataract Formation after PPV.

		Odds Ratio	95% Confidence Interval		*p*-value
**Cataract**	Age	1.86	0.34–10.28		0.466
	Refraction	0.89	0.09–7.14		0.912
	PVD	2.33	0.43–13.27		0.324
	Macula Status	7.06	1.22–58.38		0.041*
	Time	1.22	0.23–6.57		0.811
**NSC**	Age	8.25	1.52–67.18		0.024*
	Refraction	1.39	0.22–9.73		0.729
	PVD	1.50	0.32–7.66		0.612
	Macula Status	5.00	1.03–30.14		0.056
	Time	1.83	0.41–8.71		0.433
**PSC**	Age	0.84	0.18–3.98		0.823
	Refraction	0.57	0.08–3.67		0.554
	PVD	1.20	0.25–6.12		0.820
	Macula Status	3.86	0.80–22.80		0.106
	Time	1.20	0.31–6.60		0.662

Key: NSC, nuclear sclerotic cataract; PSC, posterior subcapsular cataract; PVD, posterior vitreous detachment; PPV, pars plana vitrectomy. * indicates statistically significant odds ratio

## Discussion

Oftentimes, surgical repair of RRD is surgeon dependent based on comfort, training, and experience [9]. Typically, a scleral buckle is preferred for younger patients, but as we have discussed in prior works, PPV for RRD repair has excellent outcomes for patients between 35 and 45 years of age [10–12]. Specifically, if a PVD was present prior to PPV, single surgery success rate was 87% compared to 65% without a pre-operative PVD in younger eyes [12]. In our current study, a PVD was present in 64% of eyes, but based on surgeon preference, PPV was chosen for repair in these eyes with all eyes achieving single surgery anatomic success. However, cataract formation is a well-known risk after PPV, which is significant in younger patients due to their ability to accommodate compared to older patients. Prior studies have analyzed cataract progression after retinal surgery, but only few have specifically focused on young patients or have included other pathologies such as diabetic tractional detachments and infectious and inflammatory conditions, which are inherently cataractogenic [2, 3, 13–19]. The primary purpose of this study was to analyze the rates and risk factors of cataract progression after PPV for uncomplicated RRD in healthy eyes between 15 and 45 years of age.

Within 1 year after PPV, 61% of eyes developed cataracts, which is consistent with rates reported in the literature [18]. These cataract formation rates are overall higher than the reported rates after SB alone of 24–46% [2, 20, 21]. Prior studies have demonstrated that PSC are more likely to occur after PPV in young patients, whereas NSC are most likely to occur after PPV in older patients [2, 16–18]. In our study of younger patients, NSC and PSC occurred at similar rates after PPV at 46% and 43%, respectively. While our PSC rates were similar to that reported in the literature for young eyes, our NSC rate of 46% demonstrates an interesting trend. Patients below 35 years were significantly less likely to NSC compared to patients between 35 and 45 years. This finding somewhat supports *Melberg* and *Thomas* who found minimal NSC progression in patients below 50; however, it may actually be that patients below 35 years who tend to have minimal NSC development after PPV [13]. It is possible that a younger age may be protective against NSC formation after PPV. However, when comparing all forms of cataracts, there was no significant difference in cataract formation rates in patients above and below 35 years.

Prior studies have analyzed risk factors associated with cataract progression after PPV, including refractive status, vitreous status, macula status, and surgical time [2, 3]. In our study, there was increased cataract formation after macula-off RRDs compared to macula-on RRDs. A higher rate of NSC in macula-off RRDs accounted for this difference as PSC rates were similar after both macula-on and macula-off RRDs. While this finding is difficult to explain, we suspect that this is likely due to an increased oxygen tension posterior to the lens, which has been associated with increased NSC formation [22, 23]. Prior studies have established the relationship between increased vitreous liquefaction and NSC formation as oxygen from the retinal circulation is better able to diffuse through a liquid vitreous [24]. Similarly, PPV reduces the normal oxygen gradient within the eye through multiple mechanisms including decreased vitreous oxygen consumption and increased oxygen diffusion throughout the vitreous cavity, and exposes the lens to higher oxygen concentrations [22, 25]. As the origin of subretinal fluid in a RRD is liquified vitreous, it is possible that the vitreous is more liquefied in macula-off detachments as more subretinal fluid is necessary to advance a peripheral detachment towards the macula [26]. Therefore, a thorough core and peripheral vitrectomy is able to be performed in macula-off RRDs as there is less vitreous gel. Oxygen tension posterior to the lens may be increased after a thorough vitrectomy in these cases. However, in macula-on RRDs, it is more difficult to perform a thorough vitrectomy especially in younger patients with an adherent vitreous. This may ultimately reduce oxygen tension posterior the lens due to less oxygen diffusion from the retina as well as increased vitreous oxygen consumption, and thus less cataract formation. Interestingly, in eyes with intact anterior vitreous after PPV had a significantly reduced rate of cataract formation compared to eyes without an intact anterior vitreous, which may further support this theory [27]. Additionally, *Bellucci et al.* reported increased cataract surgery rates in macula-off RRDs after PPV in all ages, which may also support our findings [14].

Prior studies have demonstrated an increased risk of cataract development with C_3_F_8_ compared to SF_6_, but these findings were not statistically significant [13, 28–31]. In our study, all but one patient had a C_3_F_8_ gas tamponade. SF_6_ gas lasts approximately 2–4 weeks in the eye, whereas C_3_F_8_ lasts approximately 2 months within the eye [32]. While eyes developing cataracts had a higher C_3_F_8_ gas fill at all time points after RRD repair up to postoperative month 2, the difference between the gas levels at these time points were not significantly different. However, all eyes with C_3_F_8_ gas at postoperative month 2 developed cataracts compared to only 59% of eyes without any residual C_3_F_8_ at postoperative month 2. While multiple factors are responsible for gas longevity including axial length, initial gas fill, and gas concentration, a shorter acting gas is likely to be less cataractogenic and should be considered in younger eyes but needs to be balanced by the higher rates of PVR and lower rates of single surgery success in this group [12, 33]. Ultimately, the decision of gas tamponade should be made to optimize primary surgical repair of RRD.

We found no other association of risk factors of cataract progression after RRD repair in young eyes. While *Pan et al*. demonstrated an association between myopia and NSC and an increased PSC rate in highly myopic patients, we did not find a similar trend in our study [34]. It has also been theorized that prolonged PPV time may predispose patients to cataract development due to increased exposure of the lens to saline solution, which was not demonstrated in our study and prior works [19, 35]. The lower rates of cataracts in eyes with intraoperative IVT use is difficult to explain as intraocular steroids has been shown to increase cataract formation [36–38]. These rates may be more attributable to the small sample sizes in each group and not a true trend after intraoperative IVT use.

The limitations of this study are its small sample size and retrospective nature. Given that this is a retrospective study, cataract grading was obtained from chart review. While majority of ophthalmologists utilize the Lens Opacities Classification System, version II (LOCS II) scale of 1–4, to grade cataracts there is also a subjective component that is difficult to standardize in a retrospective study [7]. To reduce inter-grader discrepancies, cataract grading was obtained from vitreoretinal surgeons’ documented examinations when available, but in some cases was obtained from other ophthalmologists’ examinations if grading was not available or documented by a vitreoretinal surgeon. Another limitation includes a lack of fellow eye analysis. However, as most of our cohort are young and healthy, we do not expect significant cataract progression in the fellow eye. Additionally, a longer-term follow-up may also reveal higher rates of cataract formation as they cataracts develop and progress slowly in younger patients [16].

## Conclusion

In conclusion, cataract formation was common after PPV in young patients after RRD repair with the majority of cataracts developing within 1 year of RRD repair. While our results should be interpreted with caution, our study suggests that PPV for patients below 35 years of age or macula-on RRDs may have a decreased risk for NSC formation and may be a reasonable approach for repair; however, we cannot make this statement definitively due to the sample size of this study. A shorter acting gas, such as SF_6_, should be considered in younger eyes if appropriate based on the anatomical location of retinal breaks and percentage of retinal detachment. Further prospective studies or big-data retrospective studies should be performed to better assess these risk factors for cataract formation after retinal procedures in young patients.

### Electronic supplementary material

Below is the link to the electronic supplementary material.

Supplementary Material 1

Supplementary Material 2

## Data Availability

The datasets used and/or analyzed during the current study are available from the corresponding author on reasonable request.

## Declarations

### Meeting presentation

This work was presented at the American Society for Retinal Specialists and Retina Society 2023 Annual Meeting.
