# Supplementary material for: Cataract progression after primary pars plana vitrectomy for uncomplicated rhegmatogenous retinal detachments in young adults
Source: Int J Retina Vitreous. 2024 Feb 21;10:19. doi: 10.1186/s40942-024-00538-4 (PMC10882894; doi:10.1186/s40942-024-00538-4)
Supplement: Supplementary file 1 — Supplementary Material 1 [file 40942_2024_538_MOESM1_ESM.docx]

Cataract Progression after Primary Pars Plana Vitrectomy for Uncomplicated Rhegmatogenous Retinal Detachments in Young Adults

Venkatkrish M. Kasetty, MD^1^; Pedro F. Monsalve, MD^1,2^; Dhruv Sethi, MD^1^; Candice Yousif, MD^1^; Thomas Hessburg, MD^1^; Nitin Kumar, MD^1^; Abdualrahman E. Hamad, MD^1^; Uday R. Desai, MD^1^

1. Department of Ophthalmology, Henry Ford Health System, Detroit, Michigan, USA
2. Department of Ophthalmology, University of Minnesota, Minneapolis, Minnesota, USA

**Meeting Presentation:** This work was presented at the American Society for Retinal Specialists and Retina Society 2023 Annual Meeting.

**Financial Support:** No financial support was obtained in the preparation of this manuscript.

**Conflicts of Interests:** The authors have no conflicts of interest to disclose.

**Running head**: Cataract after PPV in Young Eyes

**Corresponding Author**:

Venkatkrish M. Kasetty, MD

Henry Ford Hospital, Department of Ophthalmology

2799 W. Grand Boulevard

Detroit, Michigan 48202

Email: vkasett1@hfhs.org

Phone: (313) 676-1956

Fax: (313) 916-4757

**Abstract**

**Background:** Scleral buckling is typically implemented to repair rhegmatogenous retinal detachments (RRD) in young patients. Therefore, there is limited data on post-pars plana vitrectomy (PPV) cataract formation in this cohort. We report the rates and risk factors of cataract progression after PPV for RRD repair in young eyes. **Methods:** Retrospective single-center cohort study. Medical records of patients between the ages of 15 to 45 undergoing PPV for uncomplicated RRD between 2014 and 2020 were reviewed.

**Results:** Twenty-eight eyes from 26 patients met inclusion criteria. Cataracts developed in 20/28 (71%) eyes after PPV. After PPV, nuclear sclerotic cataract (NSC) rates were higher in patients above 35 (65%) compared to below 35 years (18%) (p = 0.024). Cataracts developed more frequently after macula-off RRDs (88%) compared to macula-on RRDs (50%) (p = 0.044) with NSC more common in macula-off detachments (p = 0.020). At postoperative month 2, all eyes with C_3_F_8_ gas developed cataracts compared to 59% of eyes with no gas (p = 0.040). **Conclusions:** Cataract formation was common and frequent after PPV. After PPV, young eyes and macula-on detachments developed cataracts less than older eyes and macula-off detachments. If appropriate, a shorter acting gas tamponade should be considered in young eyes to minimize cataract formation.

**Keywords:** Rhegmatogenous retinal detachment, vitrectomy, cataract

**Background**

Rhegmatogenous retinal detachments (RRD) occur in a bimodal distribution affecting the elderly as well as younger, typically myopic, patients.^1^ Various repair modalities exist including pars plana vitrectomy (PPV), scleral buckling (SB), and pneumatic retinopexy. PPV has been shown to increase the rate of nuclear sclerotic cataracts (NSC) and posterior subcapsular cataracts (PSC) formation with minimal lens changes seen after SB. Increased oxygen tension after vitreous removal, prolonged exposure to saline solution during PPV, use of higher purity and longer longevity gases, direct trauma to the lens, and larger vitrectomy gauges have been proposed as causes of increased cataract formation after PPV; however, the mechanism is still unclear.^2,3^ Analysis on post-PPV cataract formation is limited in young eyes as SB is usually preferred in this group.^4-6^ At our institution, some vitreoretinal surgeons prefer PPV over SB in this age group. While prior studies have evaluated the rates and risk factors associated with cataract progression after PPV in all ages, we focus our analysis on young patients to determine the rates of and risk factors for cataract progression.

**Methods**

This is a retrospective cohort study of patients ages 15 to 45 years old with no prior retinal surgery undergoing PPV for RRD repair between 2014 and 2020 at Henry Ford Health System in Michigan, USA. Primary PPV was chosen for these patients if they had a pre-operative PVD or if the vitreoretinal surgeon felt that they could induce a PVD intraoperatively. Ethical approval for this study was obtained from the Henry Ford Health System Institutional Review Board. This study adhered to the tenets of the Declaration of Helsinki. Due to the retrospective nature of the study, written informed consent was not required. Exclusion criteria included pseudophakic or aphakic eyes, eyes with less than 3 months of follow-up, eyes requiring lensectomy at the time of initial RRD repair, and combined detachments (diabetic, inflammatory, and infectious etiologies). Only eyes undergoing one surgery for RRD repair were primarily analyzed to avoid the effects of reoperations on cataract formation. Eyes with mild preoperative cataracts were included in the analysis.

Primary outcomes of this study include cataract formation rates after RRD repair and time to cataract extraction from initial PPV. Cataract formation was recorded based on type: NSC, PSC, and cortical. Cataract severity was recorded pre-operatively, at post-operative month 1 (POM1), post-operative month 3 (POM3), and post-operative year 1 (POY1). Cataract grading was based on the Lens Opacities Classification System, version II (LOCS II) scale of 1-4 (with 4 being the worst).^7^ Secondary outcomes include changes in pre- and post-cataract surgery visual acuity (VA) and correlations between age, preoperative refraction, posterior vitreous detachment (PVD) presence, macula status, surgical time and cataract development or progression. As most retinal detachments have stable VA 3-6 months after repair, postoperative month 3 was selected to minimize effects of intraocular gas and postoperative cataracts on VA in our analysis.^8^

Myopia is defined as a refraction between -6.0 and 0 diopters (D). High myopia is defined as a refraction greater than -6.0 D. Surgical time was used was a surrogate for saline infusion time during PPV.

Fisher’s exact test was performed for categorical covariates and Mann-Whitney U tests were performed for numeric covariates. All analyses were performed using RStudio statistical software (RStudio, Boston, Massachusetts, USA).

**Results**

Twenty-eight eyes (26 patients) undergoing PPV were included in this study. Initial PPV was performed by one of three vitreoretinal surgeons. The average age at PPV was 35.39 ± 8.26 years with 20/28 (71%) female. Refraction was available for 23/28 (82%) eyes, of which 8/23 (35%) and 11/23 (48%) were myopic and highly myopic, respectively. One patient (1 eye) had a diagnosis of Wagner-Stickler Syndrome and 2 patients (3 eyes) had type 2 diabetes mellitus without retinopathy. A posterior vitreous detachment (PVD) was present in 18/28 (64%) eyes with 16/28 (57%) eyes presenting with a macula-off detachment. The crystalline lens was clear in all but one eye, which had a mild cataract. Standard 23-gauge vitrectomy was performed in all vitrectomized eyes. Perfluoropropane (C_3_F_8_) gas was used in all but one vitrectomized eye where sulfur hexafluoride (SF_6_) gas was used instead. There were no documented lens touch with instrumentation during PPV. The average surgical time was 69 ± 27 minutes. Average follow-up length was 859 ± 800 days with all retinas attached at last follow-up. One eye had pre-operative proliferative vitreoretinopathy (PVR) and no eyes developed PVR after PPV. No eyes were enucleated.

Cataracts developed in 20/28 (71%) eyes after PPV with 17/28 (61%) developing within 1 year. NSC, PSC, and cortical cataracts developed in 13/28 (46%), 12/28 (43%), and 2/28 (7%) eyes, respectively, after PPV. Average cataract grading at each post-operative visit is presented in Table 1. The average NSC and PSC grading increased in PPV eyes through 1 year. NSC grading at POM1 (p = 0.021), POM3 (p = 0.011), and POY1 (p = 0.017) were significantly greater compared to pre-operative NSC grading. However, PSC grading at POM1 (p = 0.021), POM3 (p = 0.005), but not POY1 (p = 0.079) were significantly greater compared to pre-operative PSC grading. On average, cataracts developed 180 ± 308 days after PPV. Eleven (39%) eyes required cataract extraction, which occurred 375 ± 212 after PPV. The average age at PPV of eyes requiring cataract extraction was 38.27 ± 8.56 years compared to 33.52 ± 7.74 years of eyes not requiring cataract extraction after PPV (p = 0.085).

**Table 1**. Average Cataract Grading after Pars Plana Vitrectomy.

|  |  | **Pre-Operative** | **POM1** | **POM3** | **POY1** |
| --- | --- | --- | --- | --- | --- |
| **NSC** | **Grade** | 0.04 | 0.30 | 0.51 | 0.46 |
|  | **p-value** | - | 0.021* | 0.011* | 0.017* |
| **PSC** | **Grade** | 0.00 | 0.44 | 0.45 | 0.25 |
|  | **p-value** | - | 0.021* | 0.005* | 0.079 |

Key: NSC, nuclear sclerotic cataract; PSC, posterior subcapsular cataract; POM1, post-operative month 1; POM3, post-operative month 3; POY1, post-operative year one. * indicates statistically significant p-value when compared to pre-operative cataract grading.

VA changes over time are presented in Figure 1 [insert Figure 1]. After PPV, average VA improved to 20/50 from 20/100 (p = 0.513). There was a slight decrease in average VA from 20/50 to 20/60 when comparing three months after PPV to before cataract extraction (p = 0.856). The average time to cataract extraction was 375 days (range: 109-772) after PPV with 6/11 (55%) extractions occurring within 1 year of PPV. Significant VA gains were seen after cataract surgery with an improvement from 20/60 to 20/25 at the last recorded visit (p = 0.006).

Cataracts developed in 7/11 (64%) eyes in patients below 35 years compared to 13/17 (76%) of eyes in patients above 35 years (p = 0.672) (Table 2). Patients above 35 years developed NSC (11/17, 65%) more frequently than patients below 35 years (2/11, 18%) (p = 0.024). However, patients below 35 years developed PSC at similar rates (6/11, 54%) compared to patients above 35 years (7/17, 41%) (p = 0.700). Of eyes developing cataracts, 3/7 (43%) eyes below 35 years and 8/13 (62%) above 35 years required cataract extraction (p = 0.624). Cataracts also developed sooner in patients over 35 years (110 ± 147 days) compared to under 35 years (300 ± 467 days) but was not statistically significant (p = 0.140).

**Table 2**. Risk Factors for Cataract Progression after PPV.

|  |  | **Cataract Rate** | **NSC Rate** | **PSC Rate** | **Time to Cataract Development (Days)** |
| --- | --- | --- | --- | --- | --- |
| **Age** | < 35 years old | 7/11 (64%) | 2/11 (18%) | 6/11 (54%) | 300 ± 467 |
|  | ≥ 35 years old | 13/17 (76%) | 11/17 (65%) | 7/17 (41%) | 110 ± 147 |
|  | p-value | 0.672 | 0.024* | 0.700 | 0.140 |
| **Refraction** | Myopia | 6/8 (75%) | 5/8 (63%) | 4/8 (50%) | 139 ± 99 |
|  | High myopia | 8/11 (73%) | 5/11 (45%) | 4/11 (36%) | 244 ± 470 |
|  | p-value | 1.00 | 0.645 | 0.658 | 0.181 |
| **PVD** | Present | 14/18 (78%) | 9/18 (50%) | 10/18 (56%) | 230 ± 380 |
|  | Absent | 6/10 (60%) | 4/10 (40%) | 4/10 (40%) | 94 ± 71 |
|  | p-value | 0.4 | 0.695 | 0.706 | 0.902 |
| **Macula Status** | On | 6/12 (50%) | 3/12 (25%) | 3/12 (25%) | 324 ± 507 |
|  | Off | 14/16 (88%) | 12/16 (75%) | 9/16 (56%) | 114 ± 140 |
|  | p-value | 0.044* | 0.020* | 0.136 | 0.412 |
| **Surgical Time** | < 60 minutes | 9/13 (69%) | 5/13 (38%) | 5/13 (38%) | 288 ± 456 |
|  | ≥ 60 minutes | 11/15 (73%) | 8/15 (53%) | 7/15 (43%) | 102 ± 94 |
|  | p-value | 1.00 | 0.476 | 0.712 | 0.840 |

Key: NSC, nuclear sclerotic cataract; PSC, posterior subcapsular cataract; PPV, pars plana vitrectomy; PVD, posterior vitreous detachment. * denotes a statistically significant p-value.

Cataracts developed at higher rates after macula-off RRDs (14/16, 88%) compared to macula-on RRDs (6/12, 50%) (p = 0.044) (Table 2). Specifically, NSC developed more frequently in macula-off detachments (12/16, 75%) compared to macula-on detachments (3/12, 25%) (p = 0.020). There was no significant difference in PSC rates between macula-on and macula-off RRDs (p = 0.136). Cataracts also developed sooner after macula-off RRD (114 ± 140 days) compared to macula-on RRD (324 ± 507 days) but was not statistically significant (p = 0.412).

Eyes developing cataracts had an increased postoperative gas fill at all time points compared to eyes without cataracts; however, this difference was not statistically significant at any time point (Table 3). Cataracts developed in 8/8 (100%) eyes with residual C_3_F_8_ at postoperative month 2 compared to only 11/18 (61%) eyes without residual C_3_F_8_ at postoperative month 2 (p = 0.047). The eye with an SF_6_ gas tamponade did not develop a cataract.

**Table 3.** Post-PPV C_3_F_8_ Fill.

|  | **POD1 C_3_F_8_ (%)** | **POW1 C_3_F_8_ (%)** | **POM1 C_3_F_8_ (%)** | **POM2 C_3_F_8_ (%)** |
| --- | --- | --- | --- | --- |
| **Cataract** | 88.3 | 71.6 | 34.5 | 5.8 |
| **No Cataract** | 80.7 | 65.9 | 22.6 | 0 |

Key: POD1, postoperative day 1; POM1, postoperative month 1; POM2, postoperative month 2; POW1, postoperative week 1; PPV, pars plana vitrectomy.

Other risk factors for cataract development, such as myopic status, PVD presence, and surgical time was not associated with increased cataract formation rates or increased time to cataract formation (Table 2). Myopic eyes developed NSC and PSC at similar rates of 5/8 (63%) and 4/8 (50%) compared to highly myopic eyes with rates of 5/11 (45%) and 4/11 (36%), respectively (p = 0.645 and 0.658). Of eyes with a preoperative PVD, 14/18 (78%) developed cataracts compared to 6/10 (60%) eyes without preoperative PVD (p = 0.4). PSC and NSC occurred at similar rates in eyes with a PVD (56% and 50%, respectively) compared to eyes without a PVD (40% each) (p = 0.706 and 0.695). Surgical time was not associated with increased cataract formation (p = 1.00). While NSC and PSC occurred more frequently in PPV taking over 60 minutes (53% and 43%) compared to below 60 minutes (38% and 38%), there was no significant difference between the groups (p = 0.476 and 0.712). While there were no documented cases of instrument-lens touch, in eyes that may have required crossing the anatomic midline with instrumentation to treat the breaks, 11/16 (69%) developed cataracts. In eyes that did not require crossing the anatomic midline with instrumentation to treat the breaks, 9/12 (75%) developed cataracts (p=1.00). Additionally, intraoperative use of intravitreal triamcinolone acetonide (IVT) to stain the posterior hyaloid did not result in increased cataract formation. When IVT was used, 6/10 (60%) of eyes developed cataracts compared to 14/18 (78%) when IVT was not used (p=0.400). PSC occurred in 4/10 (40%) and 8/18 (44%) eyes with and without IVT use, respectively (p=1.00). NSC occurred less frequently when IVT was used 2/10 (20%) compared to when IVT was used 11/18 (61%), but was not statistically significant (p=0.055).

Univariate logistic regression analysis is presented in Table 4 and Figure 2. Similar to the non-parametric analysis above, regression analysis revealed a statistically significant correlation between macula status and overall cataract formation as well as age and NSC formation. Regression analysis did not reveal a significant association between other risk factors and cataract formation.

**Table 4**. Odds Ratios of Risk Factors Associated with Cataract Formation after PPV.

|  |  | **Odds Ratio** | **95% Confidence Interval** | | **p-value** |
| --- | --- | --- | --- | --- | --- |
| **Cataract** | Age | 1.86 | 0.34 - 10.28 | | 0.466 |
|  | Refraction | 0.89 | 0.09 - 7.14 | | 0.912 |
|  | PVD | 2.33 | 0.43 - 13.27 | | 0.324 |
|  | Macula Status | 7.06 | 1.22 - 58.38 | | 0.041* |
|  | Time | 1.22 | 0.23 - 6.57 | | 0.811 |
|  |  |  |  |  |  |
| **NSC** | Age | 8.25 | 1.52 - 67.18 | | 0.024* |
|  | Refraction | 1.39 | 0.22 - 9.73 | | 0.729 |
|  | PVD | 1.50 | 0.32 - 7.66 | | 0.612 |
|  | Macula Status | 5.00 | 1.03 - 30.14 | | 0.056 |
|  | Time | 1.83 | 0.41 - 8.71 | | 0.433 |
|  |  |  |  |  |  |
| **PSC** | Age | 0.84 | 0.18 - 3.98 | | 0.823 |
|  | Refraction | 0.57 | 0.08 - 3.67 | | 0.554 |
|  | PVD | 1.20 | 0.25 - 6.12 | | 0.820 |
|  | Macula Status | 3.86 | 0.80 - 22.80 | | 0.106 |
|  | Time | 1.20 | 0.31 - 6.60 | | 0.662 |

Key: NSC, nuclear sclerotic cataract; PSC, posterior subcapsular cataract; PVD, posterior vitreous detachment; PPV, pars plana vitrectomy. * indicates statistically significant odds ratio.

**Discussion**

Oftentimes, surgical repair of RRD is surgeon dependent based on comfort, training, and experience.^9^ Typically, a scleral buckle is preferred for younger patients, but as we have discussed in prior works, PPV for RRD repair has excellent outcomes for patients between 35 and 45 years of age.^10-12^ Specifically, if a PVD was present prior to PPV, single surgery success rate was 87% compared to 65% without a pre-operative PVD in younger eyes.^12^ In our current study, a PVD was present in 64% of eyes, but based on surgeon preference, PPV was chosen for repair in these eyes with all eyes achieving single surgery anatomic success. However, cataract formation is a well-known risk after PPV, which is significant in younger patients due to their ability to accommodate compared to older patients. Prior studies have analyzed cataract progression after retinal surgery, but only few have specifically focused on young patients or have included other pathologies such as diabetic tractional detachments and infectious and inflammatory conditions, which are inherently cataractogenic.^2,3,13-19^ The primary purpose of this study was to analyze the rates and risk factors of cataract progression after PPV for uncomplicated RRD in healthy eyes below 45 years of age.

Within 1 year after PPV, 61% of eyes developed cataracts in the groups, which is consistent with rates reported in the literature.^18^ These cataract formation rates are overall higher than the reported rates after SB alone of 24-46%.^2,20,21^ Prior studies have demonstrated that PSC are more likely to occur after PPV in young patients, whereas NSC are most likely to occur after PPV in older patients.^2,16-18^ In our study of younger patients, NSC and PSC occurred at similar rates after PPV at 46% and 43%, respectively. While our PSC rates were similar to that reported in the literature for young eyes, our NSC rate of 46% demonstrates an interesting trend. Patients below 35 years were significantly less likely to NSC compared to patients between 35-45 years. This finding somewhat supports *Melberg* and *Thomas* who found minimal NSC progression in patients below 50; however, it may actually be that patients below 35 years who tend to have minimal NSC development after PPV.^13^ It is possible that a younger age may be protective against NSC formation after PPV. However, when comparing all forms of cataracts, there was no significant difference in cataract formation rates in patients above and below 35 years.

Prior studies have analyzed risk factors associated with cataract progression after PPV, including refractive status, vitreous status, macula status, and surgical time.^2,3^ In our study, there was increased cataract formation after macula-off RRDs compared to macula-on RRDs. A higher rate of NSC in macula-off RRDs accounted for this difference as PSC rates were similar after both macula-on and macula-off RRDs. While this finding is difficult to explain, we suspect that this is likely due to an increased oxygen tension posterior to the lens, which has been associated with increased NSC formation.^22,23^ Prior studies have established the relationship between increased vitreous liquefaction and NSC formation as oxygen from the retinal circulation is better able to diffuse through a liquid vitreous.^24^ Similarly, PPV reduces the normal oxygen gradient within the eye through multiple mechanisms including decreased vitreous oxygen consumption and increased oxygen diffusion throughout the vitreous cavity, and exposes the lens to higher oxygen concentrations.^22,25^ As the origin of subretinal fluid in a RRD is liquified vitreous, it is possible that the vitreous is more liquefied in macula-off detachments as more subretinal fluid is necessary to advance a peripheral detachment towards the macula.^26^ Therefore, a thorough core and peripheral vitrectomy is able to be performed in macula-off RRDs as there is less vitreous gel. Oxygen tension posterior to the lens may be increased after a thorough vitrectomy in these cases. However, in macula-on RRDs, it is more difficult to perform a thorough vitrectomy especially in younger patients with an adherent vitreous. This may ultimately reduce oxygen tension posterior the lens due to less oxygen diffusion from the retina as well as increased vitreous oxygen consumption, and thus less cataract formation. Interestingly, in eyes with intact anterior vitreous after PPV had a significantly reduced rate of cataract formation compared to eyes without an intact anterior vitreous, which may further support this theory.^27^ Additionally, *Bellucci et al.* reported increased cataract surgery rates in macula-off RRDs after PPV in all ages, which may also support our findings.^14^

Prior studies have demonstrated an increased risk of cataract development with C_3_F_8_ compared to SF_6_, but these findings were not statistically significant.^13,28-31^ In our study, all patients except 1 had a C_3_F_8_ gas tamponade. SF_6_ gas lasts approximately 2-4 weeks in the eye, whereas C_3_F_8_ lasts approximately 2 months within the eye.^32^ While eyes developing cataracts had a higher C_3_F_8_ gas fill at all time points after RRD repair up to postoperative month 2, the difference between the gas levels at these time points were not significantly different. However, all eyes with C_3_F_8_ gas at postoperative month 2 developed cataracts compared to only 59% of eyes without any residual C_3_F_8_ at postoperative month 2. While multiple factors are responsible for gas longevity including axial length, initial gas fill, and gas concentration, a shorter acting gas is likely to be less cataractogenic and should be considered in younger eyes but needs to be balanced by the higher rates of PVR and lower rates of single surgery success in this group.^12,33^ Ultimately, the decision of gas tamponade should be made to optimize primary surgical repair of RRD.

We found no other association of risk factors of cataract progression after RRD repair in young eyes. While *Pan et al*. demonstrated an association between myopia and NSC and an increased PSC rate in highly myopic patients, we did not find a similar trend in our study.^34^ It has also been theorized that prolonged PPV time may predispose patients to cataract development due to increased exposure of the lens to saline solution, which was not demonstrated in our study and prior works.^19,35^ The lower rates of cataracts in eyes with intraoperative IVT use is difficult to explain as intraocular steroids has been shown to increase cataract formation.^36-38^ These rates may be more attributable to the small sample sizes in each group and not a true trend after intraoperative IVT use.

The limitations of this study are its small sample size and retrospective nature. Given that this is a retrospective study, cataract grading was obtained from chart review. While majority of ophthalmologists utilize the Lens Opacities Classification System, version II (LOCS II) scale of 1-4, to grade cataracts there is also a subjective component that is difficult to standardize in a retrospective study.^7^ To reduce inter-grader discrepancies, cataract grading was obtained from vitreoretinal surgeons’ documented examinations when available, but in some cases was obtained from other ophthalmologists’ examinations if grading was not available or documented by a vitreoretinal surgeon. Another limitation includes a lack of fellow eye analysis. However, as most of our cohort are young and healthy, we do not expect significant cataract progression in the fellow eye. Additionally, a longer-term follow-up may also reveal higher rates of cataract formation as they develop and progress slowly in younger patients.^16^

**Conclusion**

In conclusion, cataract formation was common after PPV in young patients after RRD repair with the majority of cataracts developing within 1 year of RRD repair. While our results should be interpreted with caution, our study suggests that PPV for patients below 35 years of age or macula-on RRDs may have a decreased risk for NSC formation and may be a reasonable approach for repair; however, we cannot make this statement definitively due to the sample size of this study. A shorter acting gas, such as SF_6_, should be considered in younger eyes if appropriate based on the anatomical location of retinal breaks and percentage of retinal detachment. Further prospective studies or big-data retrospective studies should be performed to better assess these risk factors for cataract formation after retinal procedures in young patients.

**Declarations**

*Ethics Approval and Consent to Participate*

Ethical approval for the study was obtained from the institutional review board at Henry Ford Health System (IRB # 14475). A waiver of consent was granted by the Henry Ford Health System Institutional Review Board. All data used in this study was anonymized before use. This study adhered to the tenets of the Declaration of Helsinki.

*Consent for Publication*

Not applicable.

*Availability of Data and Materials*

The datasets used and/or analyzed during the current study are available from the corresponding author on reasonable request.

*Competing Interests*

The authors have no competing interests.

*Funding*

No financial support was obtained in the preparation of this manuscript.

*Author Contributions*

VMK: conception and design of study, data collection and analysis, manuscript writing and revision

PM: data collection and analysis, manuscript writing and revision

DS: data collection, manuscript revision

CY: data collection and analysis, manuscript revision

TH: data collection, manuscript revision

NK: data collection, manuscript revision

URD: conception and design of study, data collection and analysis, manuscript writing and revision

AEH: conception and design of study, data collection and analysis, manuscript revision

All authors read and approved the final manuscript.

**Figure Legends**

**Figure 1**. Visual Acuity (VA) Changes after Intervention. After retinal reattachment, there was no significant improvement in VA compared to before PPV (p = 0.513). There was a slight decrease in VA between POM3 and pre-CS (p = 0.856). VA significantly improved after cataract surgery compared to before cataract surgery (p = 0.006). ^*^Denotes statistical significance between last recorded VA and pre-cataract surgery VA in eyes undergoing PPV. Abbreviations: CS, cataract surgery; POM3, postoperative month 3; PPV, pars plana vitrectomy.

**Figure 2**. Logistic Regression Analysis of Risk Factors Associated with Cataract Formation after PPV. ^*^Denotes statistically significant odds ratio. Abbreviations: PVD, posterior vitreous detachment; NSC, nuclear sclerosis cataract; PSC; posterior subcapsular cataract.

**References**

1. Mitry D, Charteris DG, Fleck BW, Campbell H, Singh J. The epidemiology of rhegmatogenous retinal detachment: geographical variation and clinical associations. *Br J Ophthalmol*. Jun 2010;94(6):678-84. doi:10.1136/bjo.2009.157727

2. Feng H, Adelman RA. Cataract formation following vitreoretinal procedures. *Clin Ophthalmol*. 2014;8:1957-65. doi:10.2147/opth.S68661

3. Thompson JT. The role of patient age and intraocular gas use in cataract progression after vitrectomy for macular holes and epiretinal membranes. *Am J Ophthalmol*. Feb 2004;137(2):250-7. doi:10.1016/j.ajo.2003.09.020

4. Reeves MG, Pershing S, Afshar AR. Choice of Primary Rhegmatogenous Retinal Detachment Repair Method in US Commercially Insured and Medicare Advantage Patients, 2003-2016. *Am J Ophthalmol*. Dec 2018;196:82-90. doi:10.1016/j.ajo.2018.08.024

5. Vail D, Pershing S, Reeves MG, Afshar AR. The Relative Impact of Patient, Physician, and Geographic Factors on Variation in Primary Rhegmatogenous Retinal Detachment Management. *Ophthalmology*. Jan 2020;127(1):97-106. doi:10.1016/j.ophtha.2019.04.019

6. Schaal S, Sherman MP, Barr CC, Kaplan HJ. Primary retinal detachment repair: comparison of 1-year outcomes of four surgical techniques. *Retina*. Sep 2011;31(8):1500-4. doi:10.1097/IAE.0b013e31820d3f55

7. Chylack LT, Jr., Leske MC, McCarthy D, Khu P, Kashiwagi T, Sperduto R. Lens opacities classification system II (LOCS II). *Arch Ophthalmol*. Jul 1989;107(7):991-7. doi:10.1001/archopht.1989.01070020053028

8. Ross WH. Visual recovery after macula-off retinal detachment. *Eye (Lond)*. Jul 2002;16(4):440-6. doi:10.1038/sj.eye.6700192

9. D’Amico D, Tornabe P, Edwin R. Controversies in Care: Rhegmatogenous retinal detachment repair. Pentavision. Accessed August 28, 2022. <https://www.retinalphysician.com/issues/2011/jan-feb/controversies-in-care>

10. Brown K, Yannuzzi NA, Callaway NF, et al. Surgical Outcomes Of Rhegmatogenous Retinal Detachment In Young Adults Ages 18-30 Years. *Clin Ophthalmol*. 2019;13:2135-2141. doi:10.2147/opth.S213042

11. Haugstad M, Moosmayer S, Bragadόttir R. Primary rhegmatogenous retinal detachment - surgical methods and anatomical outcome. *Acta Ophthalmol*. May 2017;95(3):247-251. doi:10.1111/aos.13295

12. Kasetty VM, Aye J, Patel N, et al. Outcomes and complications of primary rhegmatogenous retinal detachment repair with pars plana vitrectomy in young adults. *Int J Retina Vitreous*. Feb 22 2023;9(1):11. doi:10.1186/s40942-023-00448-x

13. Melberg NS, Thomas MA. Nuclear sclerotic cataract after vitrectomy in patients younger than 50 years of age. *Ophthalmology*. Oct 1995;102(10):1466-71. doi:10.1016/s0161-6420(95)30844-5

14. Bellucci C, Benatti L, Rossi M, et al. Cataract progression following lens-sparing pars plana vitrectomy for rhegmatogenous retinal detachment. *Sci Rep*. Dec 21 2022;12(1):22064. doi:10.1038/s41598-022-26415-4

15. Hernandez-Bogantes E, Abdala-Figuerola A, Olivo-Payne A, Quiros F, Wu L. Cataract Following Pars Plana Vitrectomy: A Review. *Semin Ophthalmol*. Nov 17 2021;36(8):824-831. doi:10.1080/08820538.2021.1924799

16. Kataria AS, Thompson JT. Cataract Formation and Progression in Patients Less Than 50 Years of Age after Vitrectomy. *Ophthalmol Retina*. Mar-Apr 2017;1(2):149-153. doi:10.1016/j.oret.2016.09.007

17. Thompson JT. The role of patient age and intraocular gases in cataract progression following vitrectomy for macular holes and epiretinal membranes. *Trans Am Ophthalmol Soc*. 2003;101:485-98.

18. Blodi BA, Paluska SA. Cataract after vitrectomy in young patients. *Ophthalmology*. Jul 1997;104(7):1092-5. doi:10.1016/s0161-6420(97)30180-8

19. Hosoda Y, Kuriyama S, Hattori H, Hayashi H, Matsumoto M. Outcome of primary vitrectomy in phakic patients aged younger than 50 years with rhegmatogenous retinal detachments. *Jpn J Ophthalmol*. Sep 2016;60(5):395-400. doi:10.1007/s10384-016-0463-z

20. Lv Z, Li Y, Wu Y, Qu Y. Surgical complications of primary rhegmatogenous retinal detachment: a meta-analysis. *PLoS One*. 2015;10(3):e0116493. doi:10.1371/journal.pone.0116493

21. Fallico M, Alosi P, Reibaldi M, et al. Scleral Buckling: A Review of Clinical Aspects and Current Concepts. *J Clin Med*. Jan 9 2022;11(2)doi:10.3390/jcm11020314

22. Beebe DC, Holekamp NM, Shui YB. Oxidative damage and the prevention of age-related cataracts. *Ophthalmic Res*. 2010;44(3):155-65. doi:10.1159/000316481

23. Holekamp NM, Shui YB, Beebe DC. Vitrectomy surgery increases oxygen exposure to the lens: a possible mechanism for nuclear cataract formation. *Am J Ophthalmol*. Feb 2005;139(2):302-10. doi:10.1016/j.ajo.2004.09.046

24. Harocopos GJ, Shui Y-B, McKinnon M, Holekamp NM, Gordon MO, Beebe DC. Importance of Vitreous Liquefaction in Age-Related Cataract. *Investigative Ophthalmology & Visual Science*. 2004;45(1):77-85. doi:10.1167/iovs.03-0820

25. Zong Y, Gao QY, Hui YN. Vitreous function and intervention of it with vitrectomy and other modalities. *Int J Ophthalmol*. 2022;15(6):857-867. doi:10.18240/ijo.2022.06.02

26. Popescu SI, Munteanu M, Patoni C, et al. Role of the Vitreous in Retinal Pathology: A Narrative Review. *Cureus*. Aug 2023;15(8):e43990. doi:10.7759/cureus.43990

27. Wa C, Yee K, Huang L, Sadun A, Sebag J. Long-term Safety of Vitrectomy for Patients with Floaters. *Investigative Ophthalmology & Visual Science*. 2013;54(15):2142-2142.

28. Briand S, Chalifoux E, Tourville E, et al. Prospective randomized trial: outcomes of SF_6_ versus C_3_F_8_ in macular hole surgery. *Can J Ophthalmol*. Apr 2015;50(2):95-100. doi:10.1016/j.jcjo.2014.12.006

29. Cole CJ, Charteris DG. Cataract extraction after retinal detachment repair by vitrectomy: visual outcome and complications. *Eye (Lond)*. Jun 2009;23(6):1377-81. doi:10.1038/eye.2008.255

30. Hecht I, Mimouni M, Blumenthal EZ, Barak Y. Sulfur hexafluoride (SF_6_) versus perfluoropropane (C_3_F_8_) in the intraoperative management of macular holes: a systematic review and meta-analysis. *J Ophthalmol*. 2019;2019:1820850. doi:10.1155/2019/1820850

31. Modi A, Giridhar A, Gopalakrishnan M. SULFURHEXAFLUORIDE (SF6) VERSUS PERFLUOROPROPANE (C3F8) GAS AS TAMPONADE IN MACULAR HOLE SURGERY. *Retina*. Feb 2017;37(2):283-290. doi:10.1097/iae.0000000000001124

32. Kontos A, Tee J, Stuart A, Shalchi Z, Williamson TH. Duration of intraocular gases following vitreoretinal surgery. *Graefes Arch Clin Exp Ophthalmol*. Feb 2017;255(2):231-236. doi:10.1007/s00417-016-3438-3

33. Kanclerz P, Grzybowski A. Complications Associated with the Use of Expandable Gases in Vitrectomy. *J Ophthalmol*. 2018;2018:8606494. doi:10.1155/2018/8606494

34. Pan CW, Boey PY, Cheng CY, et al. Myopia, axial length, and age-related cataract: the Singapore Malay eye study. *Invest Ophthalmol Vis Sci*. Jul 2 2013;54(7):4498-502. doi:10.1167/iovs.13-12271

35. Cheng L, Azen SP, El-Bradey MH, et al. Duration of vitrectomy and postoperative cataract in the vitrectomy for macular hole study. *Am J Ophthalmol*. Dec 2001;132(6):881-7. doi:10.1016/s0002-9394(01)01263-6

36. Chu YK, Chung EJ, Kwon OW, Lee JH, Koh HJ. Objective evaluation of cataract progression associated with a high dose intravitreal triamcinolone injection. *Eye*. 2008/07/01 2008;22(7):895-899. doi:10.1038/sj.eye.6702802

37. Islam MS, Vernon SA, Negi A. Intravitreal triamcinolone will cause posterior subcapsular cataract in most eyes with diabetic maculopathy within 2 years. *Eye*. 2007/03/01 2007;21(3):321-323. doi:10.1038/sj.eye.6702304

38. Thompson JT. Cataract formation and other complications of intravitreal triamcinolone for macular edema. *Am J Ophthalmol*. Apr 2006;141(4):629-37. doi:10.1016/j.ajo.2005.11.050
