# Supplementary material for: Cataract progression after primary pars plana vitrectomy for uncomplicated rhegmatogenous retinal detachments in young adults
Source: Int J Retina Vitreous. 2024 Feb 21;10:19. doi: 10.1186/s40942-024-00538-4 (PMC10882894; doi:10.1186/s40942-024-00538-4)
Supplement: Supplementary file 2 — Supplementary Material 2 [file 40942_2024_538_MOESM2_ESM.docx]

Antonio Marcelo Barbante Casella, MD, PhD

Gustavo Barreto de Melo, MD, PhD, FASRS

Editors-in-Chief

International Journal of Retina and Vitreous

January 25, 2024

Dear Drs. Casella and de Melo,

Thank you for considering our manuscript “Cataract Progression after Primary Pars Plana Vitrectomy for Uncomplicated Rhegmatogenous Retinal Detachments in Young Adults” for publication in the International Journal of Retina and Vitreous.

We have responded to the suggestions by the reviewers and have made edits in the manuscript accordingly. We appreciate the editorial board’s feedback and the opportunity to strengthen this manuscript. Attached is a table with our responses to the reviewers and changes that were made to the manuscript.

Thank you again for your careful revision of our manuscript and consideration for publication in the Journal of VitreoRetinal Diseases.

Sincerely,

Venkatkrish M. Kasetty, MD

Department of Ophthalmology

Henry Ford Hospital

2799 W. Grand Blvd, Detroit, MI 48202

(313)-676-1956

[vkasett1@hfhs.org](mailto:vkasett1@hfhs.org)

| Reviewer comment | Response to reviewers | Change in manuscript |
| --- | --- | --- |
| Reviewer #2: Thanks for your reply, Regarding groups i asked about, authors may add sub-grouping to their baseline data and results (as grading of cataract, type, extension of RRD, state of Ore-operative PVR, as so,  trying to rule out the risk factors for cataract progression in detail, as this is the main outcome of this article. | Thank you for the clarification. Table 2 does reference this information. Only 1 eye had a mild preoperative cataract (graded as 1+ nuclear sclerotic and 1+ cortical cataract), therefore comparing eyes with a pre-operative cataract against those without a pre-operative cataract would be difficult. RRD extension would be difficult to quantify based on just documentation and thus would involve an estimation based on the description of the RRD. Thankfully, this is indirectly analyzed in the “macula status” as a macula-on RRD would be a greater extent than a macula-off RRD. There was only one eye with pre-operative PVR and therefore we are unable to make comparisons with this single case. | Lines 106-108 edited to read: “One eye had pre-operative proliferative vitreoretinopathy (PVR) and no eyes developed PVR after PPV. No eyes were enucleated.” |
| Reviewer #3: A control group would have been interesting to compare to the study group. | Thank you for this suggestion. While a control group would be interesting, typically in the young adult age group we are analyzing, the cataract rates are minimal and multiple articles are already present regarding cataract rates in this cohort. However, comparison of cataract rates after SB were added based on rates reported in the literature as typically SB is done in the age group. | Lines 215-216 added: “These cataract formation rates are overall higher than the reported rates after SB alone of 24-46%.” |
| Between the three surgeons it would have been interesting to note any surgical approach that differed between them or if the cases also differed between them to better asses if certain cases present cataracts because of this factors. | Thank you for this inquiry. Of the cases discussed, 2 surgeons performed 27 of the cases with 1 surgeon performing only 1 of the cases. The surgical approaches were similar between all cases, however upon re-review of operative notes, there was a difference of intra-operative triamcinolone acetonide (IVT) was used between surgeries. Of the 10 eyes with IVT, 6 (60%) developed cataracts, of which 2/10 (20%) developed NS cataracts and 4/10 (40%) developed PSC cataracts. In eyes without IVT, 14/18 (79%) developed cataracts. Of these eyes, 11/18 (61%) developed NS cataracts and 8/18 (44%) developed PSC cataracts. Interestingly, there was no statistically significant difference between these groups. | Lines 182-188 added to the manuscript: “Additionally, intraoperative use of intravitreal triamcinolone acetonide (IVT) to stain the posterior hyaloid did not result in increased cataract formation. When IVT was used, 6/10 (60%) of eyes developed cataracts compared to 14/18 (78%) when IVT was not used (p=0.400). PSC occurred in 4/10 (40%) and 8/18 (44%) eyes with and without IVT use, respectively (p=1.00). NSC occurred less frequently when IVT was used 2/10 (20%) compared to when IVT was used 11/18 (61%), but was not statistically significant (p=0.055).  Lines 272-276 added to manuscript: “The lower rates of cataracts in eyes with intraoperative IVT use is difficult to explain as intraocular steroids has been shown to increase cataract formation.^36-38^ These rates may be more attributable to the small sample sizes in each group and not a true trend after intraoperative IVT use.” |
